# Molecular Dynamics Studies of the Nucleoprotein of Influenza A Virus: Role of the Protein Flexibility in RNA Binding

**DOI:** 10.1371/journal.pone.0030038

**Published:** 2012-01-17

**Authors:** Bogdan Tarus, Christophe Chevalier, Charles-Adrien Richard, Bernard Delmas, Carmelo Di Primo, Anny Slama-Schwok

**Affiliations:** 1 Virologie et Immunologie Moléculaires, UR 892, Centre INRA de Jouy en Josas, France; 2 Université of Bordeaux, ARNA Laboratory, Bordeaux, France; 3 INSERM U869, ARNA Laboratory, Institut Européen de Chimie et de Biologie, Pessac, France; University of Cambridge, United Kingdom

## Abstract

The influenza viruses contain a segmented, negative stranded RNA genome. Each RNA segment is covered by multiple copies of the nucleoprotein (NP). X-ray structures have shown that NP contains well-structured domains juxtaposed with regions of missing electron densities corresponding to loops. In this study, we tested if these flexible loops gated or promoted RNA binding and RNA-induced oligomerization of NP. We first performed molecular dynamics simulations of wt NP monomer and trimer in comparison with the R361A protein mutated in the RNA binding groove, using the H1N1 NP as the initial structure. Calculation of the root-mean-square fluctuations highlighted the presence of two flexible loops in NP trimer: loop 1 (73–90), loop 2 (200–214). In NP, loops 1 and 2 formed a 10–15 Å-wide pinch giving access to the RNA binding groove. Loop 1 was stabilized by interactions with K113 of the adjacent β-sheet 1 (91–112) that interacted with the RNA grove (linker 360–373) via multiple hydrophobic contacts. In R361A, a salt bridge formed between E80 of loop 1 and R208 of loop 2 driven by hydrophobic contacts between L79 and W207, due to a decreased flexibility of loop 2 and loop 1 unfolding. Thus, RNA could not access its binding groove in R361A; accordingly, R361A had a much lower affinity for RNA than NP. Disruption of the E80-R208 interaction in the triple mutant R361A-E80A-E81A increased its RNA binding affinity and restored its oligomerization back to wt levels in contrast with impaired levels of R361A. Our data suggest that the flexibility of loops 1 and 2 is required for RNA sampling and binding which likely involve conformational change(s) of the nucleoprotein.

## Introduction

The nucleoprotein of Influenza A virus, NP, covers and protects the eight single-stranded viral RNA segments of negative polarity [1], [2], [3], [4], [5], [6], [7], [8], [9], [10], [11]. NP assembles with the three subunits of the polymerase into a ribonucleoprotein complex (RNP) which controls transcription and replication. NP has a key role in this complex, regulating the balance between transcription and replication during the virus cycle [6], [8], [10]. The sequence of NP is highly conserved among virus types and subtypes.

Recently, X-ray structures have shown that NP forms a trimer in the crystalline state [7], [11]. The subunit interactions in the trimer were mediated by a swapping tail loop. In particular salt bridges between adjacent monomers were essential for the stability of the trimer; the single point mutation located in the swapping loop, R416A, disrupted the trimer by breaking a salt bridge with E339 of the adjacent monomer and R416A exclusively formed monomers. In the H5N1 structure, the trimer interface was stabilized by an additional salt bridge between K430 of one subunit and E434 of the neighbor subunit [7], [11]. The trimer interface also presented hydrophobic patches conferring further stabilization through π−π stacking and hydrophobic interactions [12]. Each NP monomer within the trimer is organized into the head domain, the body domain and the tail loop region. The head and body domains are well structured and have a high helical content. Some of these helices present a sequence conservation of 80 to 100%. In between the head and body domains, a protruding element (167–186) and a basic loop (72–91) surround a concave groove, rich in basic residues, mostly arginines which likely constitutes the RNA binding site. The electron density in the basic loop was missing in the X-ray structures [7], [11], [13], [14] but the importance of this loop for RNA binding was shown by a substantial decrease of the affinity for RNA of the deletion mutant NP-Δ74–88 compared to NP [7]. Residues R74, R75 of loop 1 and R174 and R175 of the protruding element were found essential for RNA binding [7].

In this study, we questioned the role of the flexible elements found in NP structure in promoting RNA binding and oligomerization. To that end, we quantified the flexibility of NP by molecular dynamics (MD) simulations, based on the X-ray structure of the H1N1 protein to which the missing regions have been added. Molecular modeling is an available tool to study protein dynamics, especially of flexible regions unsolved by X-ray crystallography, without the need of labeling high protein concentrations required for NMR studies. The flexibility of the wt protein was compared to the fluctuations of a mutant R361A selected for its location in the RNA binding groove. If the flexibility of the loop regions impacts on the RNA binding groove and mediates RNA binding and protein self-association, differences should be observed between NP and R361A. The simulations highlighted the flexibility of three defined regions in NP monomer that were perturbed either by oligomerization via trimer formation or by the R361A mutation. The first two regions were the basic loop 1 and the protruding element (loop 2) on one face of the protein and the oligomerization loop 3 on the other face of the protein. The flexibility of loops 1 and 2 facilitated RNA binding to wt NP, presumably mediated by a conformational change of these loops as previously proposed [15]. In contrast, a limited access for RNA binding was seen in R361A, caused by a reduced loop flexibility via a salt bridge between E80 (loop 1) and R208 (loop 2) and hydrophobic interactions between loops 1 and 2 observed in the dynamic simulations. To test this hypothesis, we expressed wt NP, wt-E80A-E81A and wt-R204A-R208A on the one hand and the R361A and the triple mutants R361A-E80A-E81A, R361A-R204A-R208A on the other hand. The replacement of the two consecutive glutamates or R204A and R208A by two alanines aimed at avoiding the formation of the salt bridge between loops 1 and 2 and at recovering access for RNA binding. The affinity of these proteins for RNA was determined by surface plasmon resonance and their RNA-induced oligomerization was monitored by dynamic light scattering.

## Results

### Molecular Dynamics simulations

We tested (1) the existence of flexible elements included in NP structure and (2) the putative role of these flexible regions in promoting RNA binding and NP oligomerization. To that end, we used NP in both monomer and trimer forms. The simulations first analyzed one NP monomer, based on the available PBD file (2IQH [11]) (see experimental methods). The single-point mutation R361A, located in the RNA binding groove, was created from the wt structure and used for testing the relationships between flexibility, RNA binding and RNA-induced oligomerization. The wt monomer and R361A mutant were simulated over runs of 50 ns for each protein in explicit solvent conditions to insure correct electrostatic interactions in these highly charged proteins. We calculated the root-mean-square fluctuations (RMSF) of the protein backbone during the dynamics run to identify flexible regions in a quantitative way. We choose to represent the fluctuations of the backbone atoms because they are representative of the secondary structures without interference of the interactions between side-chains and solvent.

#### Comparison between the fluctuations of NP monomer and trimer

**Figure 1:**
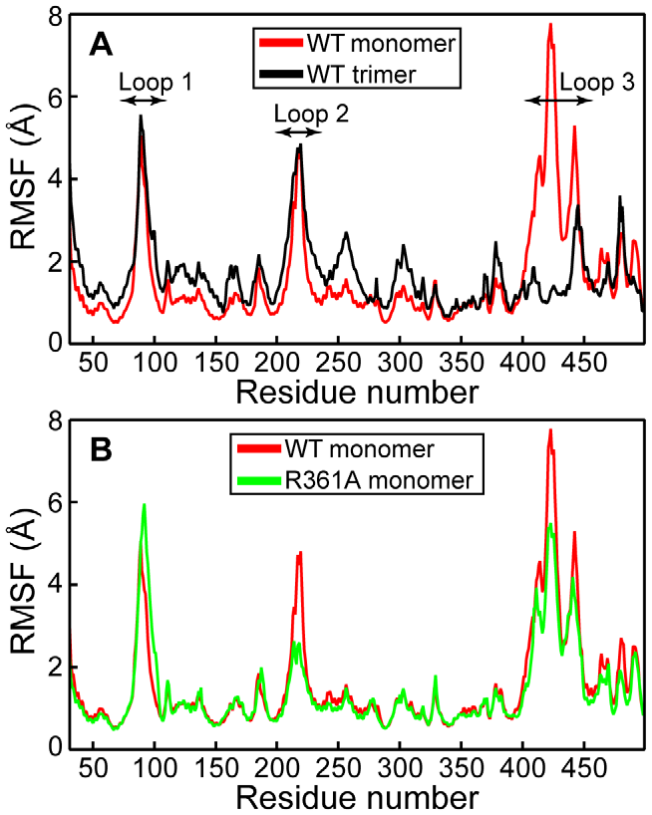
Comparison of the NP and R361A proteins by molecular modelling. **A:** Comparison the flexibility of loops 1 and 2 in the trimer (black) and monomer (red) forms of NP quantified by their backbone root-mean-square fluctuations during 4 ns and 50 ns simulation time, respectively. Loop 1 (73–90) and loop 2 (200–214) remained flexible in both NP forms; in contrast, a large difference is seen in the oligomerization loop 3 (402–428) of NP monomer and trimer. **B:** Root-mean-square fluctuations of the NP (red) and R361A (green) monomers during the simulated trajectories: one can see a reduced flexibility in loop 2 and a small increase of the flexibility of loop 1 of the R361A mutant.

**Figure 2:**
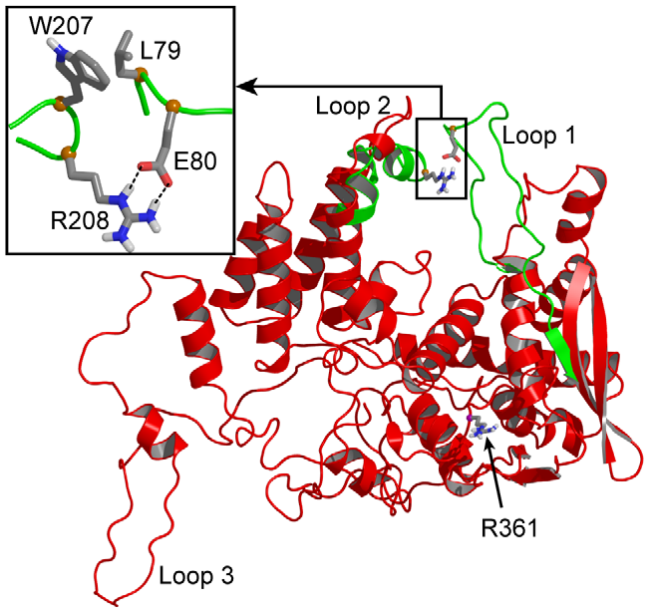
Comparison of representative structures of NP (red) and the R361A mutant (green) proteins. The position of the mutated residue R361 is highlighted in CPK representation; the salt bridge between residues E80 and R208 and hydrophobic interactions between L79 and W207 stabilized the relative positions of the two loops at shorter distance in R361A than in NP (insert).

The backbone RMSF of NP trimer and of NP monomer were calculated and compared. Figure 1A shows the existence of three flexible regions in NP monomer which displayed a significantly high RMSF. These regions corresponded to flexible loops, the first two encircling the RNA binding groove, loop 1, also called basic loop (73–90) and loop 2 (200–214) (Figure 2). The third loop corresponded to the oligomerization domain protruding in the neighbor protomer within the trimeric structure (402–428). The flexibility of loops 1 and 2 in each protomer of the NP trimer was very similar to that seen in NP monomer in isolation. This result allowed at extending the simulation time by reducing the size of the system from trimer to monomer. A large difference of flexibility between the oligomerization loop 3 of the trimer compared to that of the monomer was found. The RMSF value of loop 3 dropping near zero in the trimer is consistent with its good electron density observed in the crystal structure. In contrast, the large flexibility of the oligomerization loop could be expected by the lack of protein-protein interactions in the NP monomer. Interestingly, the flexibility of the C-terminus of NP trimer was lower than the N-terminus. Indeed, the F488 and F489 of the C-terminus were buried within a hydrophobic area in NP trimer.

#### Comparison between the fluctuations of NP and R361A monomers

The RMSF value decreased markedly in loop 2 while it increased somewhat in loop 1 of R361A compared to loops 1 and 2 of NP monomer respectively (Figure 1B). In R361A, the flexibility of loop 3 also presented a relative decrease. Figure 2 highlights the large structural difference between NP and R361A in the relative position of loops 1 and 2. A representative structure of the NP is shown in red. Loops 1 and 2 encircle the RNA binding domain in which the R361 residue is located, forming a pinch that has to be wide enough to accommodate RNA (Figure 2). In NP, loop 1 was partly structured (see below). Figure 2 shows the superimposed structure of the loops 1 and 2 of the R361A mutant represented in green on NP monomer structure. Loop 1 became unstructured in R361A and came in close contact with loop 2, stabilized by a salt bridge between E80 and R208 (Figure 2, inset). The formation of the salt bridge is consistent with the RMSF decrease of loop 2 in R361A relative to that of NP (Figure 1B). The small increase RSMF of loop 1 is likely due to increased interactions with loop 2 (see below). L79 of loop 1 made hydrophobic interactions with W207 and the aliphatic portion of R208 (carbons C_β_ and C_γ_) of loop 2 which drove and stabilized the loop-loop interactions in R361A. Thus, the smaller size of the “pinch” formed by loops 1 and 2 that may hold the RNA in place and the lower flexibility of loop 2 reduced the accessibility of the RNA binding groove in the mutant as compared to NP. Indeed, R361A has a lower affinity for RNA and a reduced ability to oligomerize (see below).

**Figure 3:**
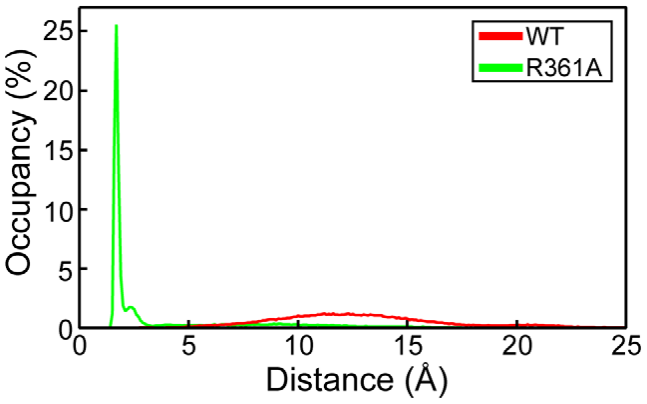
Distribution of the minimal distance between loops 1 and 2 in NP and the R361A mutant. It characterized the differences in loops interactions between wt NP and R361A.

To characterize the interactions between loops 1 and 2, we calculated the minimal distance between loops 1 and 2 observed in the wt and mutated proteins during the dynamics (Table 1). In the NP, the aperture distance was maximal, ranging between 10 and 15 Å, large enough for RNA binding (Figure 3). In contrast, this distance was drastically reduced to a value of 2.5 Å in the R361A mutant, consistent with the formation of strong interactions bridging the two loops, in agreement with the formation of a salt bridge and hydrophobic contacts (Figure 2).

**Table 1:** Analysis of the interactions observed in four domains of the wt NP and R361A mutant.

Contact domains	Contact residues	Contact type	WT contact population (%)	R361A contact population (%)
a) loop1-loop2	L79-loop2	HPh^2^	0	67
key contacts	(R204 or R208)-(E80 or E81)	HB^1^	0	60
b) loop1 base	K113-loop1	HB^1^	99	45
Stability	K113-E73	HB^1^	68	23
c) linker-β sheet 1 contacts	Y97-M371	HPh^2^	76	73
	R106-linker backbone	HB^1^	77	63
	K103-E372	HB^1^	65	38
d) inter-linker	R317-E369	HB^1^	73	93
Contacts	R361-E369	HB^1^	51	-

Loop 1 (residues 73–90), loop2 (residues 200–211), β-sheet 1 (residues 91–112), and the linker (residues 360–373).

**Figure 4:**
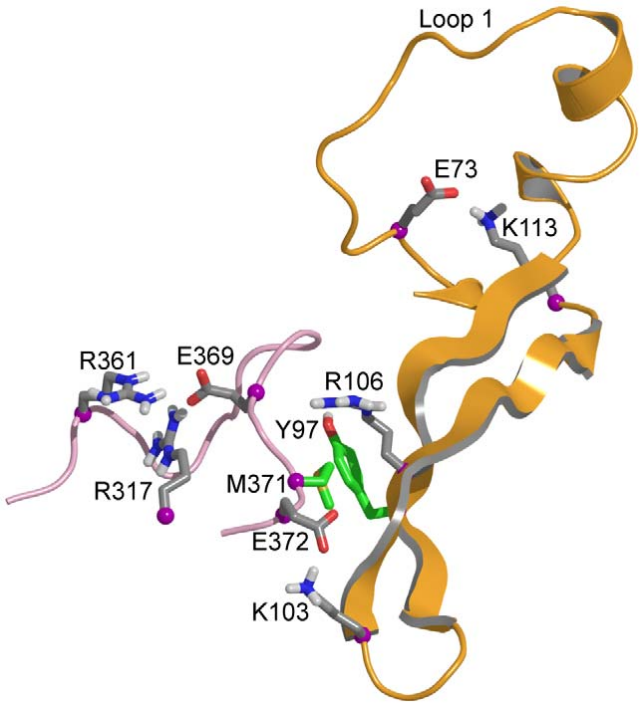
Proposed communication path between loop 1 and the RNA groove. K113 located at the edge of β-sheet 1 strongly interacts with loop 1, in particular E73. The other extremity of the β-sheet had multiple contacts with residues of the RNA grove, in particular hydrophobic interactions between Y97 and M371, interactions between R106 and the linker backbone (residues 360–373 shown in magenta) and electrostatic interactions between K103 and E372. The linker itself was stabilized by salt bridges between E369 and R361 and E369 and R317 (Table 1).

These interactions define a path between loop 1 and the residue R361 of the linker, located in the RNA binding groove (see Figures 2 and 4).

1 HB stands for hydrogen bond.

2 HPh stands for hydrophobic interaction.

See the Experimental section for definitions of the contact domains and the two contact types.

a) Loop 1-loop 2 contact: In R361A, hydrophobic interactions between L79 and loop 2 drove loop 1 to contact loop2; transient salt-bridges between R204 or R208, on the one hand, and E80 or E81, on the other hand, stabilized the interaction between loop 1 and loop 2 at short distances. Such loop-loop interactions were not found in wt NP.

b) Loop 1 stability (base): The side-chain of K113 was engaged in a strong hydrogen bond with the C-terminus of the loop 1; the guanidinium moiety of K113 formed a salt bridge with E73 at the N-terminus of loop 1, contributing to the stability of loop 1 in wt NP. The R361A mutation drastically reduced the interactions of the K113 with the loop 1, increasing loop 1 flexibility.

c) Linker-β sheet 1 contacts: The linker was connected to the β sheet 1 through conserved hydrophobic interaction between M371 and the ring of Y97, stable hydrogen bonds between R106 and the linker backbone oxygen atoms. The R361A mutation decreased the population of the solvated K103-E372 salt-bridge.

d) Inter-linker contacts: In NP, E369 interacted with both R361 and R317. In R361A, the R317-E369 contact population increased to 93% as compared to 73% in wt NP and the R361-E369 interaction was canceled by the mutation.

#### Detailed analysis of loop 1 and a proposed model for communication between loop 1 and the RNA groove in NP

In NP, loop 1 was stabilized by a salt bridge between K113, located at the end of the first β-sheet and E73 at the basis of loop 1 (Figure 4 and Table 1). K113 also contacted the backbone oxygen atoms of P89 and K90. The cumulated stabilizing interactions of K113 with loop 1 counteracted the destabilizing effect of P83 and P89 at C-terminus of loop 1; proline residues are usually destabilizing an alpha helix. In all trajectories, K113 always interacted with loop 1 residues as shown in Table 1 (interaction populated 99%). K113 is located at the end of the first β-sheet. The other extremity of this β-sheet was connected with the RNA groove residue R361 located on a rigid linker (defined by residues 360–373), mediated by multiple interactions detailed in Figure 4. Hydrophobic interactions between the aromatic ring of Y97 and M371 of the linker, electrostatic interactions between K103 and E372, interactions between R106 and the linker were often observed. A salt bridge between R361 and E369 of the linker and sometimes of E369 and R317 rigidified the linker. We hypothesized that a rigid linker is required for transmission of the information between the RNA groove via the β-sheet to loop 1 (Table 1). We found a strong correlation, 0.75, between the fluctuations of the linker (the principal axes of the moment of inertia of residues 360–373) and of the loop fragment (backbone atoms of residues 110–113). This correlation supported the proposed path of communication between the linker in the RNA groove and loop 1 through the mediation of β-sheet 1.

#### Analysis of loop 1 in R361A

Figure 2 shows the superimposed structure of the loops 1 and 2 of the R361A mutant represented in green on NP monomer structure shown in red. Loop 1 became unstructured in R361A. The salt bridge K113-E73 was less populated than in NP (Table 1). Transient interactions between E73 and R174 were found in this mutant (data not shown). Consequently, loop 1 of the R361A mutant was unstructured and more elongated than in NP (30.6 Å and 37.6 Å for wt NP and R361A respectively) which allowed loop-loop contacts.

In conclusion, the MD simulations suggested that the flexibility of loops 1 and 2 of NP monomer may be required to grasp RNA and promote RNA binding. This feature was also seen in the trimeric form of NP (Figure 1B). The flexibility of loop 2 was reduced by the R361A mutation, located on a linker of the RNA binding domain. This reduced flexibility was caused, at least partly, by interactions between loops 1 and 2, mediated by a salt bridge between E80 and R208 and additional hydrophobic interactions (Figure 2), thus the gate for RNA accessing its binding groove was narrowed from 12 Å in NP to 2.5 Å in R361A (Figures 2, 3).

To test this model, we expressed the recombinant NP, R361A, wt- E80A- E81A and R361A- E80A-E81A proteins. We determined their RNA binding affinity and oligomerization. The mutation of the two consecutive aspartates E80 E81 into alanines should abolish the salt bridge between E80 and R208 (without the possibility of a compensating interaction between E81 and R208) and facilitate access to the RNA binding groove. We expected improved RNA binding and RNA-induced oligomerization in this triple mutant as compared to that in R361A.

### Characterization of RNA-free wt NP, wt-E80A-E81A, wt-R204A-R208A, R361A and R361A-E80A-E81A, R361A-R204A-R208A mutants

The wt NP protein and the R361A, wt-E80A-E81A, wt-R204A-R208A and R361A-E80A-E81A, R361A-R204A-R208A mutants were expressed as C-terminal His-tagged proteins in *E. coli* and purified after RNAse treatment by affinity and size-exclusion chromatographies (data not shown). Using low salt conditions (50 mM NaCl), NP eluted in a main peak at (81.6±1.0) ml. NP was eluted at a similar retention volume than R416A mutant, known to be monomeric [2], [3], [4], [5], [11], [15]. The R361A mutant was eluted into two main peaks at 81.6 ml and 70.4 ml, resembling the chromatographic profile of NP, but required 300 mM NaCl for elution from the size-exclusion column. The double and triple mutant eluted in a main peak that corresponded to monomeric species. MALDI-TOF analyses further confirmed that these bands were NP and its mutants. The maximal absorption at 280 nm in each preparation attested the absence of nucleic acid contaminants in NP and mutants preparations. Once separated, NP remained in a monomeric form when kept at 50 mM NaCl and 4°C, however purifications under higher salt conditions resulted in oligomer formation as previously reported [7], [11]. Analytical ultra-centrifugation further confirmed that the proteins were mainly monomeric (data not shown).

#### Characterization of the affinity of NP and the loop 1 mutants for RNA

**Figure 5:**
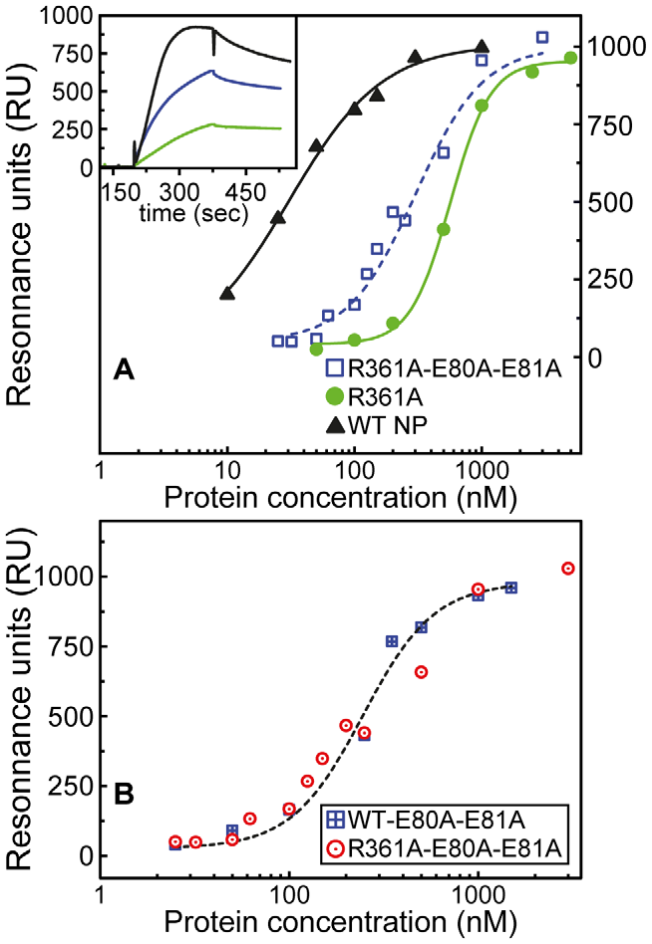
Influence of mutations in loop 1 of NP on RNA binding. **A:** Effect of the mutations R361A (green circles) and R361A-E80A-E81A (blue squares) compared to wt NP (black triangles) binding to RNA; Inset: comparison of the SPR signals obtained in the presence of Flu1-RNA with 300 nM C-terminal His-tagged NP, R361A or R361A-E80A-E81A. Due to its low affinity for RNA, the signal of the R361A-RNA complex (green) is ca four times smaller than NP-RNA (black), while the signal of the triple mutant (blue) is intermediate between them. The binding of NP or mutants to the surface-bound Flu1-RNA oligonucleotide followed a saturation curve with maximal RU at large protein concentration; the signal deduced from the plateau of the association kinetics as a function of NP concentration was used to obtain the Kd, taken as the concentration at which the RU is 50% of the maximal RU. **B:** Binding to Flu1-RNA of the double and triple mutants, wt-E80A-E81A (blue squares), R361A-E80A-E81A (red circles) respectively.

Surface plasmon resonance experiments were carried out to analyze the binding kinetics of NP and its mutants to single-stranded linear RNA. The 24-mer RNA fragment used for this experiment had the same 24-mer sequence, named Flu1, which was used in previous studies [7], [11]. Typical results are presented in Figure 5. The insert of Figure 5 shows that the association of NP to RNA resulted in a fast NP-RNA complex, in contrast with the slower association of the R361A mutant to RNA yielding to a ca three times lower signal using a protein concentration of 400 nM. This mainly resulted from a lower association rate constant for the R361A monomer compared to that of the wt. In contrast, the signal of the R361A-E80A-E81A triple mutant was about twice larger than that of R361A. The stoichiometry of the complex was determined by comparing the expected binding signal of the NP-RNA complex (expected molecular weight of the complex MW = 57000+7574) to the observed signal due to the hybridization of a short oligonucleotide complementary to the Flu1-RNA sequence (MW = 2388) [16]; the complex formed between monomeric NP and RNA displayed a 1∶1 stoichiometry.

Figure 5 also shows that the signals due NP binding to RNA (recorded at the end of the association phase) increased with the increase of the protein concentrations at low nM NP concentrations, and then reached saturation around 300 nM. The resonance unit corresponding to 50% of the saturation plateau was taken as the apparent dissociation equilibrium constant Kd. The apparent equilibrium dissociation constants were found to be Kd = 41±7 nM, 240±26 nM and 565±60 nM for NP, R361A-E80A-E81A and R361A respectively. The triple mutant recovered part of the affinity for RNA that was lost in the single mutant R361A. This result comforts the hypothesis of the salt bridge between R208 and E80 in R361A that reduced access to RNA for its binding groove, leading in R361A to an increase of the apparent Kd (loss of affinity) and a decrease of the association rate constant to RNA compared to NP (Figure 5A, insert). We also tested the E80A E81A mutation in the context of wt NP: its affinity for RNA was identical within experimental error to that observed with the triple mutant Kd = 280±40 nM (Figure 5B). Therefore, the remaining affinity difference between R361A-E80A-E81A and wt NP was due to the E80A-E81A mutation without contribution of the R361A single mutation.

The monomeric RNA-free proteins formed NP/RNA complexes of 1/1 stoichiometry, showing that RNA binding precedes NP oligomerization. The oligomerization was followed by dynamic light scattering (DLS) that monitored the change of the hydrodynamic diameter of NP as a function of time after the addition of RNA.

#### Kinetics of oligomerization of monomeric NP monitored by DLS

**Figure 6:**
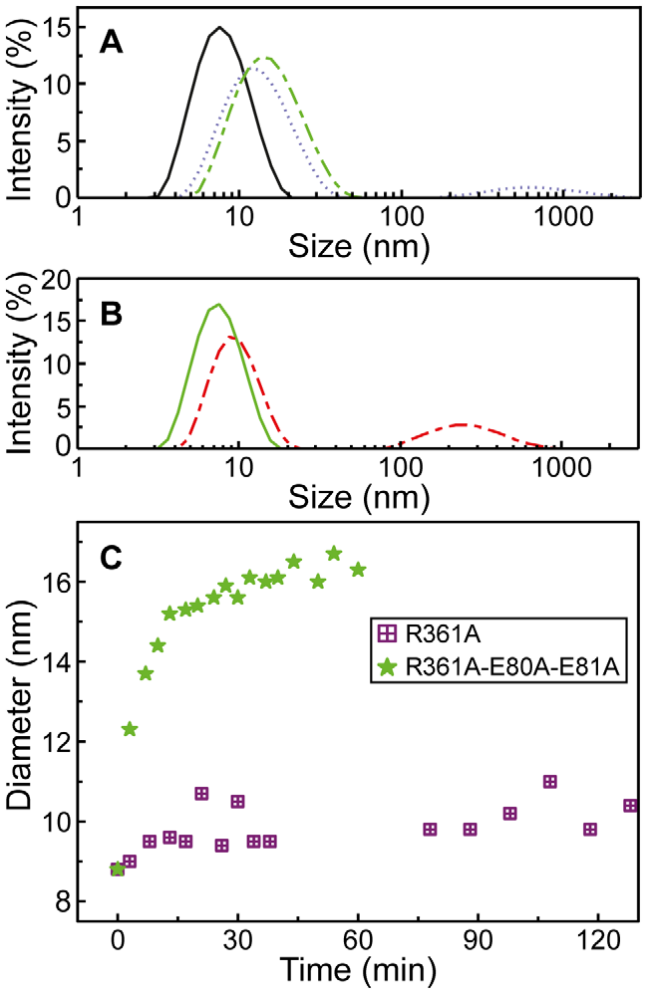
Size of NP oligomers in the presence of RNA monitored by Dynamic Light Scattering. **A** - Size distribution of monomeric wt NP (5 µM) alone (black, 6.8 nm, 93%, 5.0 µ, 7%) and 1 hour (dotted blue, 13.8 nm 91%, 760 nm 9%) or 3 hours after addition of 1.8 µM RNA (16.3 nm, 100%); **B**- Size distribution of monomeric R361A (5 µM) alone (black, 7.8 nm, 100%) and 4 hours after addition of 1.8 µM RNA (dashed green, 9.85 nm, 75%, 279 nm, 25%) **C**: Comparison of the oligomerization kinetics of R361A (violet squares) and R361A-E80A-E81A (green stars) (10 µM) after addition of RNA (3 µM). Note the large difference in the final size of the protein-RNA oligomers being 10±1 nm and 16±1 nm for R361A and R361A-E80A-E81A, the latter resembling the size of oligomeric NP-RNA complexes.

Unlabeled RNA oligonucleotides were used to determine the apparent size of the NP-RNA complexes. Upon addition of RNA, an increase of the diameter of free NP from D_h_ = 7±1 to 16±1 nm was observed and corresponded to RNA-induced oligomerization (Figure 6A). The size of the NP - RNA oligomers was comparable to that formed with wt-E80A-E81A in the presence of RNA (data not shown). The extent of RNA-induced oligomerization of R361A was lower than that of NP: once formed, R361A-RNA oligomers had an apparent smaller size of D_h_ = 11±1 nm than D_h_ = 16±1 nm observed for the NP-RNA oligomers (Figure 6A and B). In contrast, the triple mutant R361A-E80A-E81A formed oligomers of D_h_ = 16±2 nm, recovered an oligomerization rate similar to that of NP, in contrast with R361A-RNA oligomers (Figure 6C).

#### Comparison between the loop 1 and loop 2-mutants

**Figure 7:**
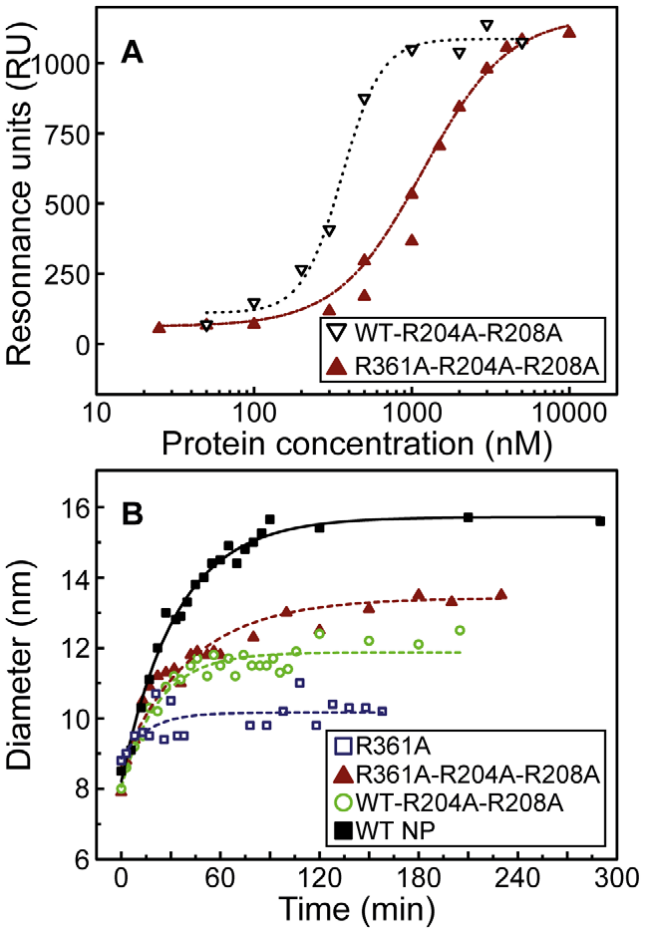
Influence of mutations in loop 2 of NP on RNA binding and RNA-induced oligomerization. **A:** Comparison of the association to and dissociation from RNA of R361A-R204A-R208A (full triangles) and wt-R204A-R208A (open triangles); **B:** Comparison of the oligomerization kinetics of 10 µM proteins after addition of RNA (3 µM): wt NP (full squares), R361A (open squares), wt-R204A-R208A (open circles) and R361A-R204A-R208A (full triangles) (10 µM). The lines represent single exponential fits.

The wt- R204A- R208A protein in which the mutation belongs to loop 2 had a RNA binding affinity of Kd = 350±40 nM, close to Kd = 280±40 nM observed for the wt-E80A-E81A mutant. When compared to Kd = 41±7 nM, the data showed the importance of both loops 1 and 2 in RNA binding (Figure 4b and 7a). In contrast with the ability of the loop 1-double mutant to form oligomers with an apparent diameter of D_h_ = 16±1 nm as NP did, wt- R204A- R208A oligomers had a smaller size of D_h_ = 12±1 nm (Figure 7b and data not shown). These data show the involvement of loop 2 in oligomerization.

The affinity of the R361A-R204A-R208A mutant for RNA was lower than that of R361A, Kd = 1.1±0.1 µM and 565±60 nM, respectively. However, the ability to form RNA-NP oligomers of normal size was partly recovered in R361A-R204A-R208A for which the oligomers had a diameter of D_h_ = 14±1 nm, compared to D_h_ = 11±1 nm observed in the presence R361A.

## Discussion

NP adopted in solution multiple oligomeric forms in equilibrium with monomers, while trimers were found in the crystalline state [7], [11]. Trimers were assumed to be non physiological species; the assembly of NP nonamers with the polymerase complex was solved by electronic microscopy [17], highlighting the importance of the oligomerization process in NP function. NP crystal structures present areas with low electronic density corresponding to loops. We build these loops with the SWISS-MODEL package and added them to one NP monomer, using the published structure (PDB 2IQH) [11]. The energy of the system placed in explicit solvent was minimized and stabilized before productive dynamics runs. RMSF measurement of the protein during the dynamics identified highly flexible regions compared with regions of low RMSF found in α-helices in NP [11]. The flexible regions in NP trimer were the basic loop 1 (73–90) and loop 2 (200–214). NP monomer had a third flexible loop (402–428) that corresponded to the swapping loop used for stabilization of the trimeric structure (Figure 1).

In NP, loops 1 and 2 formed a pinch of 10 to 15 Å, wide enough to accommodate RNA (Figures 2, 3). The C-terminus of loop 1, between P83 and P89, was partly structured in α helical portions, as proline is known to be an α -helix breaker [18]. A key residue stabilizing loop 1 of NP was K113 protruding in loop 1 from the edge of β-sheet 1 (Figure 4). In the X-ray structure, the residues of loop 1 were not solved but K113 was oriented (by an interaction of its aliphatic part with A70) toward the gap between D72 and T92 exactly at the expected position of loop 1. This orientation of K113 supported the hypothesis that K113 tended to burry in loop 1. The importance of K113 was previously suggested by the inability of the K113A mutant to rescue viral growth. K113A did not induce significant change in RNP function [4] and the mutant K90A, K91A, K113A, R117A, R121A had the same affinity for RNA than wt NP [7], suggesting compensation of the K113A mutation (by other charged residues) for RNA binding.

We hypothesized that the flexibility of these loops had a function in RNA capture and these loops may be closing up around RNA when present (Figures 1 and 2). Our modelling was performed in the timescale of a few ns and cannot probe other fluctuation motions that would take place more slowly. We run MD simulations of NP trimer placed in explicit solvent during 4 ns. Figure 1A shows that both loops 1 and 2 remained flexible as found in NP monomer. We further tested loops flexibility in the R361A protein, the mutated residue being located in the RNA binding groove and carried by a linker (360–373). In R361A, loop 1 elongated as compared to that in NP and the interactions of K113 with loop 1 were weaker (Table 1). The minimal distance between the tips of loops 1 and 2 was shortened (Figure 3) and a salt bridge between E80 and R208 formed, driven and stabilized by hydrophobic interactions of adjacent residues, in particular L79 and W207 (Figure 2). In loop 2, a marked decrease of the flexibility as compared to that of NP was observed in R361A (Figure 1A). This suggested hindrance to RNA sampling and a decreased accessibility to the RNA groove of this mutant that may subsequently affect oligomerization. Nevertheless, it seems likely that W207, being located at the tip of loop 2, could contribute to RNA binding by hydrophobic interactions with RNA base(s) in this mutant. R361A had a markedly decreased affinity for RNA compared to that of NP, Kd = 565 nM and 41 nM respectively. The value of Kd = 41 nM obtained for monomeric NP is in agreement with previous reports [12], [19], [20].

We assumed that the affinity drop of R361A compared to wt NP was due to the formation of a salt bridge between loop 1 (E80) and loop 2 (R208) and expressed the mutant R361A-E80A-E81A in an attempt to disrupt the putative salt bridge E80-R208. As expected, this triple mutant had a Kd of 240 nM for RNA as compared to 565 nM for R361A (Figure 5). In addition, the Kd of the triple mutant and the double mutant wt-E80A-E81A were similar. The resulting ca. six fold affinity loss compared to wt NP corresponded to the effect of the E80A-E81A mutation in loop 1, supporting the hypothesis that loop 1 was involved in RNA sampling and/or capture. Deletion of the 74–88 residues of loop 1 indeed decreased the affinity for RNA by 5.25 fold [7].

We also expressed loop 2 mutants. The double mutation R204A-R208A aimed at avoiding a compensating salt bridge formation between R204 and E80 or E81. The wt-R204A-R208A mutant exhibited a decrease of ca. eight fold compared to the Kd of wt NP, similarly to the affinity loss of wt-E80A-E81A. However in R361A-R204A-R208A, the protein–RNA interactions were weaker than in R361A. We assume that the reduced flexibility of loop 2 (Figure 1B) and its increased hydrophobicity helped maintaining transient contacts with loop 1 in R361A-R204A-R208A despite the rupture of the E80-R208 salt bridge, explaining its low affinity for RNA. The reduction of the electrostatic interactions by mutation of R208A and R204A at the tip of loop 2 likely will enhance hydrophobic contacts between L79 and W207 (Figure 2 and Table 1). Together with compensatory hydrophobic interactions between L79 and A208 and A204 in loop 2- triple mutant, a hydrophobic patch may be established at the tip of loop 2, maintaining transient interactions between E73 and R174 and between R74, R75 and E210 of loop 2, R74, R75, R174 and R175 being essential for RNA binding [7]. In contrast, in loop -1 triple mutant, W207 could not establish hydrophobic contacts with A80 and A81 and the recovery of RNA-binding and oligomerization was facilitated by the larger fluctuation movements of loop 1 enhancing RNA sampling (Figure 1B and 2).

The R361A-R204A-R208A protein formed oligomers of significantly larger size than those observed with R361A, while R361A-E80A-E81A oligomers were as large as to wt ones within experimental error, Figures 6C and 7B. Thus, the expected rupture of the E80 – R208 salt bridge by mutation improved RNA-induced oligomerization as compared to that observed in R361A. Interestingly, the wt-R204A-R208A mutant was unable to generate oligomeric species of the same size as NP and the wt-E80A-E81A did. These data clearly suggested a role of loop 2 in NP oligomerization, in agreement with the largely decreased transcription/replication efficiency of the RNP complex and loss of function [4] by the R208A mutation. Moreover, loop 2 was characterized as part of the bipartite NLS which was shown to be essential for viral replication [11], [21], [22]. Thus, a single point mutation, in particular in these loops or in their vicinity as in the RNA groove, may easily shift the NP (folding) energy and affect the path of the NP-NP interactions.

Altogether, the drastic increase of the Kd for RNA and the subsequent altered oligomerization of R361A relative to NP may be mediated by a reduced flexibility of loop 2 and hindrance to access to its RNA binding groove and its vicinity. The R361A mutation did not alter RNA polymerase activity although the virus could not be rescued [4] which suggested that the RNP compensated the effect of the R361A mutation: for example, NP oligomerization rate could be enhanced by one or more protein of the polymerase complex.

The simulations taken together with the experimental data have suggested that flexible loops 1 and 2 are required for NP activity. Movements of loops 1 and 2 could be part of the NP conformational changes induced by RNA binding. We cannot exclude that additional conformational changes may take place in NP in longer timescales. It is likely that the basic loop 1 helps sampling the environment as proposed [7], [15]. The oligomerization process of the double mutant wt-E80A-E81A was similar to that of the wt, suggesting that loop 1 was not involved in NP oligomerization. Once RNA enters the cavity between the two loops, E80 and E81 of loop 1 may confine RNA in the binding groove by electrostatic repulsion. This suggests that the double mutation may affect RNA dissociation from NP, in agreement with the dissociation rate constants calculated from the SPR data, being k_off_ = 0.004 s^−1^ for the NP-RNA complex as compared to k_off_ = 0.007 s^−1^ for the wt-E801-E81A-RNA complex. The importance of the basic loop 1 can be also deduced from epitope mapping of a monoclonal antibody directed against NP [23] that recognized specifically the 71–96 region; another antibody bound to the 1–162 region of NP of 15 Influenza A subtypes [23]. In conclusion, this loops flexibility deduced from the X-ray structures and studied in this work seems important for NP function and could be exploited by cellular or viral factors to modify or regulate NP activity.

## Methods

### Molecular dynamics (MD) simulation and data analysis

The MD simulations of the NP, R361A monomers and NP trimer were carried out using the program NAMD [24] with the CHARMM27 force field [25]. The crystal structure of H1N1 influenza A virus nucleoprotein (PDB ID: 2IQH [11]) was used as an initial configuration. The missing 3D coordinates were added using the SWISS-MODEL package [26]. The NP was solvated in an explicit molecular water model TIP3P [27]. The NP and R361A monomers and NP trimer were centered in a box of pre-equilibrated water molecules with edge of 120 Å and 155 Å, respectively. The monomer and trimer solvated systems were electrostatically neutralized by adding 16 (15 for the R361A mutant) and 48 chloride ions, respectively, at points of minimal electrostatic energy. The ionic strength of the solution was set to 0.15 M by adding ions of sodium and chloride at random coordinates in solution. The van der Waals interactions were smoothly shifted to zero between 10.0 Å and 12.0 Å. The list of the non-bonded interactions was truncated at 13.5 Å. The lengths of the bonds containing hydrogen atoms were fixed with the SHAKE algorithm [28] and the equations of motion were iterated using a time step of 2 fs in the velocity Verlet integrator. The electrostatic interactions were calculated with no truncation, using the particle mesh Ewald summation algorithm [29]. The energy of the system was minimized during 5000 steps using the conjugate gradient energy minimization algorithm while the solute atoms were harmonically restraint to their initial positions with a force constant of 50.0 kcal/mol/Å^2^. The system was heated linearly to 300 K over 60 ps. Molecular dynamics simulation in NPT ensemble was used to equilibrate the system and for production run. During the 1.6 ns equilibration phase, the restrains applied of the solute atoms were gradually reduced from 5.0 kcal/mol/Å^2^ to zero. The pressure and temperature were restrained to 1 atm and 300 K, respectively. Five trajectories of 10 ns each were produced for the wt NP and R361A monomers, respectively. One trajectory of 4 ns was simulated for the NP trimer. The simulated trajectories were further analyzed using the molecular dynamics simulation and the analysis package CHARMM [30].

*Root-mean-square fluctuation (RMSF:.* Elimination of translation and rotation of protein configurations generated during the simulations was initially performed. The root-mean-square displacement (RMSD) of the protein atoms from averaged positions was further minimized and averaged over the residue backbone heavy atoms.

*Direct correlation function* of two vectors, **A** and **B** was calculated using <**A**(0)·**B**(t)>.

### Contact definition

An hydrogen bond was considered to form when the distance between the donor and acceptor heavy atoms was less than 3.5 Å. An interaction between two hydrophobic residues was considered if there is a space between their side-chains which cannot accommodate one water molecule.We estimated that the minimum distance between heavy atoms of two hydrophobic side-chains should be less than 6.1 Å.

### Chemicals and oligonucleotides

NaCl (99.9% purity), Tris-HCl, glycerol were purchased from Sigma. The RNA oligonucleotides were synthesized on an Expedite 8909 synthesizer and purified by electrophoresis on denaturating acrylamide gels or purchased at Eurofins MWG Open with HPLC purification. The oligonucleotides immobilized on streptavidin sensor chips were biotinylated at their 5′ end. The sequences of the oligonucleotides are listed below:

Flu-1: 5′ UUU GUU ACA CAC ACA CAC GCU GUG 3′

rU25: 5′ UUU UUU UUU UUU UUU UUU UUU UUU U 3′

### Protein expression & purification of NP, R361A, the wt-E80A-E81A, wt-R204A-R208A and R361A-E80A-E81A, R361A-R204A-R208A mutants

We cloned the full-length nucleoprotein NP gene of the H1N1 (strain A/WSN/33) with a 6-His-tag at its C-terminus end in the pET22 vector (Novagen) under the control of a T7 promotor. The R361A single mutation and R361A-E80A-E81A, R361A-R204A-R208A triple mutations were introduced by using *Pfu* DNA polymerase with the QuickChange site-directed mutagenesis kit (Stratagene). Nucleotide sequencing was carried out to confirm the presence of the nucleotide substitution. The *Escherichia coli* BL21 (DE3) cells carrying the plasmids were induced 4 hours by isopropyl-β-D-thiogalactopyranoside (IPTG) at 37°C (NP) or 12 h at 28°C (R361A, the double mutant wt-E80A-E81A and the triple mutant R361A-E80A-E81A) and collected by centrifugation. The pellet was resuspended and sonicated in a lysis buffer (20 mM Tris at pH = 7.4 with NaCl (50 mM or 300 mM), 5 mM imidazole, 1% Triton and 1 mg/ml lysozyme) and treated with 0.15 mg/ml RNAse A at 35°C for 20 minutes in the presence of 10 mM Mg^2+^. The proteins were purified by IMAC-Ni^2+^ affinity chromatography followed by size-exclusion chromatography. The NP was purified at 50 mM NaCl (in this condition, NP was mainly monomer). The R416A, R361A, the double mutants wt-E80A-E81A, wt-R204A-R208A and the mutants triple R361A-E80A-E81A, R361A-R204A-R208A were purified at 300 mM NaCl. We used a Superdex S200 column with an enhanced separation for molecular weights in the range 15 to 100 KDa. After purification, the protein concentration was determined by the extinction coefficient ε = 56200 M^−1^.cm^−1^ at 280 nm.

### Surface plasmon resonance experiments

The binding kinetics were performed on a Biacore 3000 apparatus using streptavidin coated sensorchips (SA, Biacore) prepared as indicated by the manufacturer. Immobilization of the biotinylated oligonucleotide on the streptavidin coated sensorship was carried out in PBS [7], [11]. The oligonucleotides were denatured at 80°C and renatured slowly at room temperature for one hour before each experiment. To reduce the non-specific response to minimal values, 300 mM NaCl and 0.025% P20 surfactant were added to the running buffer containing 300 mM NaCl, 20 mM Tris-HCl buffer, pH = 7.4. Measurements were conducted at 25°C and samples were injected at 25 µl/min flow rate. In each experiment, NP was first injected to the immobilized RNA probe; this association phase was followed by the dissociation of the NP-RNA complex after buffer injection. One flowcell left blank was used as a reference. The NP and mutant proteins were injected at concentrations between 4 and 1000 nM (NP), and 20 and 5000 nM (E361A and wt-E80A-E81A and R361A-E80A-E81A). A short oligonucleotide complementary to part of the biotinylated Flu1 probe was used for calibration of the signal of the NP-RNA complex on each sensor chip.

### Dynamic light scattering

The measurements were performed on a Malvern nanosizer apparatus thermostated at 20°C. The size distribution was calibrated with latex particles of 65 and 200 nm radius before the determination of the apparent hydrodynamic size. The scattering intensity data were processed using the instrumental software to obtain the hydrodynamic diameter (*D*_h_) and the size distribution of scatters in each sample. Hydrodynamic diameters of the particles were estimated from the autocorrelation function, using the Cumulants method. In a typical size distribution plot from the DLS measurement, the *x* axis shows a distribution of size classes (nm) and the *y* axis shows the relative intensity of the scattered light. A total of 10 scans with an overall duration of 5 min were obtained for each sample and time point. All data were analyzed in triplicate. The protein concentrations usually were in the range of 5 to 15 µM. The oligomerization was conducted at 20°C in 50 mM NaCl, 20 mM Tris at pH = 7.5.

### Analytical ultracentrifugation

The experiments were performed at a concentration of 15 and 20 µM of NP or its R416A and R361A mutants using an XLA70 ultracentrifuge (Beckman Coulter, Palo Alto, USA), equipped with an ANTi-60 rotor. The ultracentrifugation was performed at 45 000 rpm (147 280 g), 15°C. The absorption at 280 nm was recorded every 5 min and 55 scans were averaged. The data were analyzed using the Sedfit and Svedberg softwares [31], [32].
